# Phosphorylation of p53 Serine 15 Is a Predictor of Survival for Patients with Hepatocellular Carcinoma

**DOI:** 10.1155/2019/9015453

**Published:** 2019-02-07

**Authors:** Taewoo Yang, Yegyun Choi, Jae Won Joh, Steve K. Cho, Dae-Shick Kim, Sung-Gyoo Park

**Affiliations:** ^1^School of Life Sciences, Gwangju Institute of Science and Technology, Gwangju 61005, Republic of Korea; ^2^Department of Surgery, Samsung Medical Center, Seoul 06351, Republic of Korea; ^3^Department of Pathology, Samsung Medical Center, Samsung Biomedical Research Institute, Sungkyunkwan University School of Medicine, Seoul 06351, Republic of Korea

## Abstract

**Background:**

Hepatocellular carcinoma (HCC) is one of the most common malignant cancers with a poor prognosis. Several commonly investigated immunohistochemical markers in resected HCC have potential prognostic value, but the prognostic utility of p53 expression in HCC has remained elusive.

**Aim:**

To evaluate the prognostic value of p53 and p53 phosphorylation at serine 15 (p53 Ser15-P) in patients with HCC.

**Methods:**

Surgically resected tumors from 199 HCC patients were analyzed for p21, p53, p53 Ser15-P, and proliferating cell nuclear antigen (PCNA) expression using immunohistochemistry.

**Results:**

Stratifying by the expression of p53 Ser15-P (*P *= 0.016), but not by p53 (*P* = 0.301), revealed significantly different survival outcomes in patients with HCC. Moreover, our analysis demonstrated that patients who were PCNA-positive and p53 Ser15-P–negative had significantly worse survival outcomes (*P *= 0.001) than patients who were PCNA-positive and p53 Ser15-P–positive.

**Conclusions:**

P53 Ser15-P is associated with poor outcomes in patients with HCC, and this prognostic marker is useful for predicting the survival of patients with PCNA-positive HCC.

## 1. Introduction

Despite many scientific and clinical advances, the malignant cancer hepatocellular carcinoma (HCC) has a high rate of mortality [1]. Generally, HCC is classified into four differentiation grades according to Edmondson's and Steiner's classification: well differentiated (grade I), moderately differentiated (grade II), poorly differentiated (grade III), and undifferentiated (grade IV) [2]. HCC dedifferentiation correlates with increasingly aggressive features including proliferation, invasion, distant metastasis, and treatment resistance [3].

p53 has an antitumorigenic effect because it controls the cell cycle, apoptosis, or both [4]. It is also involved in tumor cell invasion [5, 6]. p53 protein levels are usually very low in normal cells, but when cells are confronted with stressors, such as ultraviolet radiation, reactive oxygen species (ROS), ionizing radiation, or oncogenic activation, p53 becomes stabilized by posttranslational modifications and accumulated in the cells [4, 7]. Because of its essential role as a tumor suppressor, p53 is called the “guardian of the genome”. In HCC patients' livers, there are more cells than in normal livers because the tumor cells proliferate imprudently. This condition produces more ROS in the livers of HCC patients, and this ROS induces DNA damage in the liver cells [8]. This could explain why p53 protein levels increase in HCC. The most important function of p53 is the transactivation of target genes. One of the most important p53 target genes is* p21*, which is critical for cell-cycle arrest and the induction of apoptosis under stressful conditions [9, 10]. p21 is a nuclear protein that induces cell-cycle arrest in the G1 and G2 phases by inhibiting cyclin/cyclin-dependent kinase complexes and the function of proliferating cell nuclear antigen (PCNA) [11, 12]. Clinical studies have shown that p21 serves as a prognostic factor for HCC patient survival [13]. However, the relationship of p53 to the survival of HCC patients is still controversial although p53 is related to p21 [14–17].

In addition to the stabilization of p53, phosphorylation at different sites of p53 also modulates p53 transcriptional activity [7]. Among the various phosphorylation sites of p53, phosphorylation in the transactivation domain (TAD) is the most important for transcriptional activity [7, 18]. Under genotoxic conditions, the expression of the ataxia telangiectasia-mutated and Rad3-related protein kinases is essential for p53 TAD phosphorylation at serine 15 (p53 Ser15-P) [19, 20]. In addition, other kinds of kinases, including casein kinase 1, AMP-activated protein kinase, and DNA-dependent serine/threonine protein kinase, are also involved in TAD phosphorylation under different kinds of stressful conditions such as starvation and oncogene activation [20]. p53 TAD phosphorylation at serine 15 is primary response to DNA damage, and it is major response for p53 activation as the phosphorylation stimulates association of p53 with histone/lysine acetyltransferases (HATs), which is important for p53 stabilization and activation [20, 21].

In this report, we analyzed the expression of p53, p53 Ser15-P, p53 phosphorylation at Ser392 (p53 Ser392-P), p21, and PCNA in 199 HCC patients. We found that p53 Ser15-P was linked to p21 protein expression. In addition, patients who had Ser15 phosphorylated p53 had a higher overall survival rate than patients who did not. Furthermore, this tendency was present in PCNA-positive HCC patients, but not in PCNA-negative patients. Thus, our data suggest that p53 Ser15-P can be used as a prognostic factor for HCC patients, especially PCNA-positive patients.

## 2. Patients and Methods

### 2.1. Patients

The specimens (n=199) were obtained from patients at the Samsung Hospital, Sungkyunkwan University College of Medicine, Seoul, Korea, between December 2000 and December 2005. Institutional Review Board of Sungkyunkwan University approved the tissue collection and this study. In 199 patients, 97 patients had liver cirrhosis, and 102 patients did not have liver cirrhosis. The tumor stages were evaluated according to the* AJCC Cancer Staging Manual*, 6^th^ edition. Of the 199 patients, 82 had T1 stage HCC tumor, 84 had T2 stage HCC tumor, and 33 had T3 stage HCC tumor. All the patients are monitored for the survival analysis until 22 January 2012 (follow-up period: 2-89 months; patients status (live/death): 154/45).  Table 1 summarizes detailed information of the tumor characteristics.

### 2.2. Immunohistochemistry

p53, p53 Ser15-P, p53 Ser392-P, p21, and PCNA were examined by immunohistochemistry (IHC) on formalin-fixed, paraffin-embedded sections after xylene deparaffinization and alcohol rehydration. Antigen retrieval was achieved by microwaving in 10 mmol/L sodium citrate buffer at pH 6 for 12 min. Endogenous peroxidase was quenched by incubating the slide with 3% H_2_O_2_ for 10 min. After washing, slides were incubated in saturating solution (PBS containing 1% BSA) for 30 min at room temperature (RT), followed by a 1 h incubation at RT with primary antibodies against p53 (ab4060, Abcam), p53 Ser15-P (ab38497, Abcam), p53 Ser392-P (ab33889, Abcam), p21 (#2947, Cell Signaling), and PCNA (ab15497, Abcam). After washing, slides were incubated for 30 min at RT with biotinylated anti-rabbit secondary antibody (E0432, Dako S.p.A) and for 30 min at RT with HPR-streptavidin. The peroxidase reaction was developed with 3, 3′-diaminobenzidine (Dako), and sections were counterstained with hematoxylin. Slides incubated with secondary antibody alone were used as negative controls.

### 2.3. Staining Evaluation

For statistical analysis, expression levels of p53, p53 Ser15-P, p53 Ser392-P, p21, and PCNA were classified semiquantitatively based on scores of positive-staining tumor cell percentage. The percentage of positive cells was scored as “0” if <1% of the tumor cells were stained positive, “1” if 1-4% were stained positive, “2” if 5-19% were stained positive, “3” if 20-69% were stained positive, and “4” if 70-100% were stained positive. This scoring is used in statistical analysis. For Kaplan-Meier Curve analysis, receiver operating curve (ROC) was used to establish each cut-off value for p53, p53 Ser15-P, p53 Ser392-P, p21, and PCNA.

## 3. Statistical Analysis

We compiled the statistics of the 199 HCC patients. Statistical analyses were performed using the Statistical Package for the Social Sciences software ver.12 (SPSS Inc.) and Origin software ver.9.1 (OriginLab Corporation). Correlations between factors are calculated with Spearman's rank correlation analysis because it is a better method for ordinal variables. A* P* value of less than 0.05 was considered to be statistically significant. A* P* value of less than 0.01 was considered to be highly significant. Correlation between molecular factors and clinicopathological factor was also calculated with Spearman's rank correlation analysis. Overall survival curve (OS) was drawn with Kaplan-Meier survival analysis. In this analysis, patients who are dead in 5-year period are indicated as decrease of graph. Patients who are cured in 5-year period are indicated as censoring. A* P* value of less than 0.05 was considered to be statistically significant. A* P* value of less than 0.01 was considered to be highly significant. Multivariate analysis was done with Cox proportional hazard analysis. Cox proportional hazard analysis was done with demographic factors (age and gender) and clinical factors (p53, p53 Ser15-P, p53 Ser392-P, and PCNA).

## 4. Results and Discussion

### 4.1. p53 and p53 Ser15-P Expression Are Correlated with p21 and PCNA Expression, but p53 Ser392-P Expression Is Not

**Figure 1:**
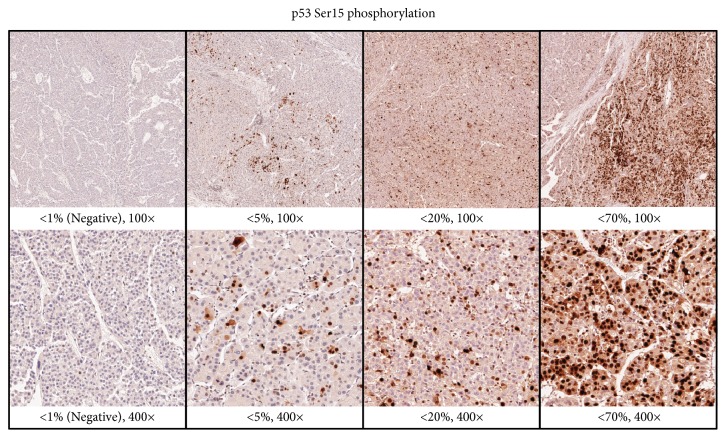
*Expression of p53 Ser15-P as observed by IHC*. Brown dots are the stained p53 Ser15-P–positive cells. Categories of <1%, 5%, 20%, and 70% positive cells (PP) are shown under 100× and 400× magnification.

TAD phosphorylation, especially p53 Ser15-P, is important for transcriptional activation [7, 18, 20, 21]; thus we hypothesized that p53 TAD phosphorylation at serine 15 would play an important role in HCC progression and prognosis. We analyzed the Spearman's rank correlation between p21 and p53 Ser15-P, or p53 Ser392-P in 199 HCC patients (Tables 1 and 2, and Figure 1). p53 Ser392 is not located in TAD of p53 [22, 23]. Thus, to test the relationship of TAD-unrelated p53 phosphorylation site with HCC progression and prognosis, p53 Ser392-P was used. Correlation coefficient between p53 Ser15-P and p21 (0.309) was higher than correlation coefficient between p53 Ser392-P and p21 (0.018) (Table 3). Correlation between p53 Ser15-P and p21 was highly significant (*P* ≤ 0.001) (Table 3), but correlation between p53 Ser392-P and p21 was not (*P* = 0.801) (Table 3). Next, we analyzed the Spearman's rank correlations between p21, p53, and p53 Ser15-P. We found that correlation coefficient between p53 Ser15-P and p21 (0.309) was higher than correlation coefficient between p53 and p21 (0.191) (Table 3). But, both correlations were highly significant (*P* ≤ 0.001 and* P* = 0.007, respectively) (Table 3). This demonstrated that unlike p53 Ser392-P, both p53 expression and p53 Ser15-P play an important role in p21 expression.

Because PCNA was known as strong biomarker of HCC [24], correlation between p53 Ser392-P, p53 Ser15-P, and PCNA was also checked. In this data, correlation coefficient between PCNA and p53 Ser15-P (0.239) was higher than correlation coefficient between PCNA and p53 Ser392-P (0.100) (Table 3). Correlation between p53 Ser15-P and PCNA was highly significant (*P* = 0.001) (Table 3), but correlation between p53 Ser392-P and PCNA was not (*P* = 0.162) (Table 3). This suggested a possibility that p53 Ser15-P is more reliable with survival than p53 Ser392-P.

### 4.2. p53 Serine 15 Phosphorylation Is Not Correlated with HCC Clinicopathological Features but Correlated with 5-Year Survival

p21 is a well-known protein that prevents CDK2-cyclin E complex formation by combining with CDK2 to stop the cell cycle (from G1 to S) when the cell has critical problems, and it serves as prognostic factor for HCC patient survival [11–13]. In the above data, we found that p53 Ser15-P is significantly correlated with p21 expression and also with PCNA which is strong biomarker of HCC (Table 3). Based on this, we hypothesized that p53 Ser15-P would correlate with progression of HCC and we analyzed the Spearman's rank correlation between clinicopathological factors and p53 Ser15-P. Unexpectedly, p53 Ser15-P did not correlate with clinicopathological features such as vascular invasion (*P* = 0.888), major portal vein invasion (*P* = 0.599), and intrahepatic invasion (*P* = 0.323) (Table 4). However, p53 Ser15-P correlated with 5-year survival (*P* = 0.023). p53 expression and p53 Ser392-P both were not correlated with 5-year survival (*P* = 0.373 and* P* = 0.873, respectively) (Table 4). PCNA was highly correlated with vascular invasion (*P* = 0.003), major portal vein invasion (*P* = 0.002), intrahepatic invasion (*P* ≤ 0.001), and 5-year survival (*P* = 0.004) (Table 4), as previously reported [24]. These results indicated that p53 Ser15-P played different roles in the progression of HCC.

### 4.3. p53 Serine 15 Phosphorylation Can Be Used as a New Prognostic Factor for HCC

**Figure 2:**
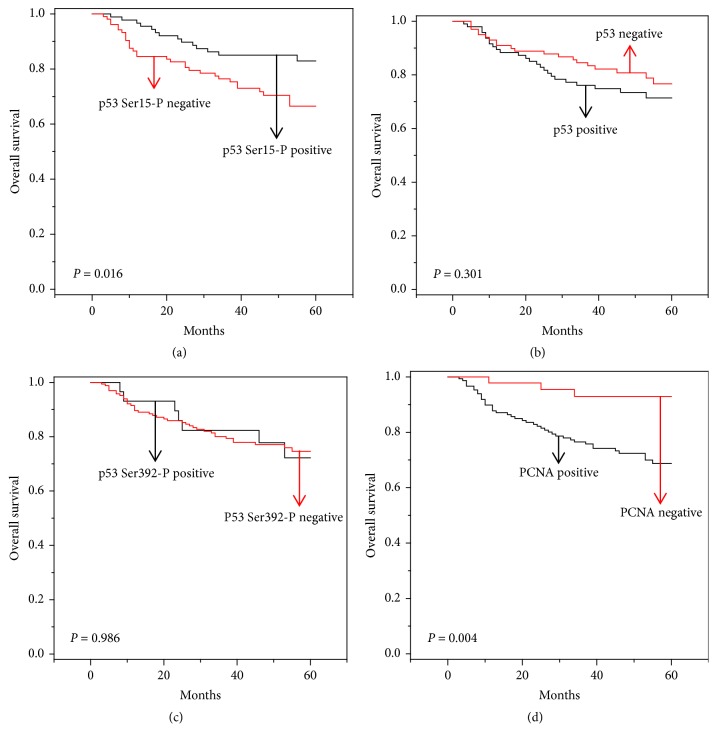
*Five-year overall survival (OS) curves of HCC patients with positive or negative expression of p53, p53 Ser15-P, p53 Ser392-P, and PCNA by Kaplan-Meier Curve*. Black lines are the positive individuals, and red lines are the negative individuals. (a) OS graph of p53 Ser15-P–positive and –negative individuals. (b) OS graph of p53-positive and -negative individuals. (c) OS graph of p53 Ser392-P–positive and –negative individuals. (d) OS graph of PCNA-positive and -negative individuals.* P*,* P* value from log rank tests.

**Figure 3:**
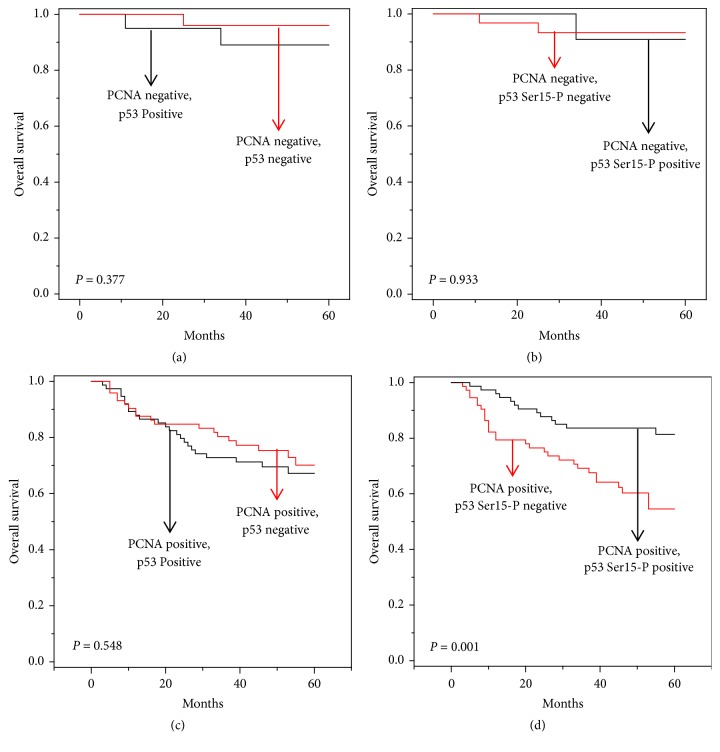
*Five-year overall survival (OS) curves of PCNA-positive or –negative HCC patients with positive or negative expression of p53, and p53 Ser15-P by Kaplan-Meier Curve*. (a, b) OS graphs of the PCNA-negative individuals. (a) OS graph of PCNA-negative and p53-positive and -negative individuals. Black line is p53 positive, and red line is p53 negative. (b) OS graph of PCNA-negative and p53 Ser15-P–positive and –negative individuals. Black line is p53 Ser15-P–positive and red line is p53 Ser15-P–negative. (c, d) OS graphs of PCNA-positive population. (c) OS graph of the PCNA-positive and p53-positive and -negative individuals. Black line is p53 positive, and red line is p53 negative. (d) OS graph of the PCNA-positive and p53 Ser15-P–positive and –negative individuals. Black line is p53 Ser15-P–positive, and red line is p53 serine 15-P negative.* P*,* P* value from log rank tests.

In our data, we found that p53 Ser15-P correlated with the expression of p21 (Table 3), but not with clinicopathological factors (Table 4). When we made a Kaplan-Meier survival curve, however, we found a correlation between p53 Ser15-P and survival rate. When we drew separate Kaplan-Meier survival curves for the p53 Ser15-P–positive/negative individuals and p53-positive/negative individuals, we detected a higher number of survivors and a correlation with the 5-year survival rate in the p53 Ser15-P–positive patients (*P*= 0.016) (Figure 2(a)), but not in the p53-positive patients (*P* = 0.301) (Figure 2(b)) and p53 Ser392-P–positive patients (*P* = 0.986) (Figure 2(c)). PCNA had a high correlation with overall survival (*P* = 0.004) as previously reported [24] (Figure 2(d)). Next, we combined p53 Ser15-P and PCNA and analyzed the correlation between survival rate and p53 Ser15-P in PCNA-positive and -negative individuals. If p53 Ser15-P could predict survival in PCNA-positive and/or -negative patients, then p53 Ser15-P could be used to determine a more precise HCC prognosis. In the PCNA-negative individuals, there was no correlation between 5-year survival and p53 expression (*P* = 0.377) (Figure 3(a)) or p53 Ser15-P (*P* = 0.933) (Figure 3(b)). In the PCNA-positive individuals, there was no correlation between 5-year survival and p53 expression (*P* = 0.548) (Figure 3(c)), but there was a correlation between 5-year survival and p53 Ser15-P (*P* = 0.001) (Figure 3(d)). In addition, we performed the Cox proportional hazard model analysis, which is a kind of multivariate analysis (Table 5). In this analysis, p53 Ser15-P (Exp(B) = 0.614;* P* = 0.013), PCNA (Exp(B)* =* 1.608;* P* = 0.002), age (Exp(B) = 1.093;* P* ≤ 0.001), vascular invasion (Exp(B)* =* 3.977;* P* = 0.006), major portal vein invasion (Exp(B)* =* 6.289;* P* ≤ 0.001), and intrahepatic invasion (Exp(B)* =* 3.823;* P* ≤ 0.001) showed significant correlation with 5-year survival of patients (Table 5). But, p53 (Exp(B) = 0.904;* P* = 0.398), p53 Ser392-P (Exp(B) = 0.925;* P *= 0.696), and sex of patients (Exp(B) = 1.085;* P* = 0.853) did not show correlation with 5-year survival of patients (Table 5). Thus, our data suggested that p53 Ser15-P could be considered as prognostic factor for HCC patients.

It has been known that p53 had antitumorigenic effect [4], but our study showed the expression of p53 could not be used for predicting survival of HCC patients as prognostic value of p53 expression in HCC was low. Our analysis showed the significant statistical correlation of p53 Ser15-P with p21 and PCNA expression. Thus, we hypothesized that there could be correlation between p53 Ser15-P and prognosis of HCC patient survival.

To check this hypothesis, we analyzed the relationship between p53 Ser15-P and clinicopathological factors. From the analysis, p53 Ser15-P was not statistically correlated with any of the clinicopathological factors. However, when we examined statistical correlation between p53 Ser15-P and 5-year survival, we found significant correlation between 5-year survival and p53 Ser15-P. This may imply that p53 TAD phosphorylation is important for antitumorigenic effect of p53. Moreover, our data suggest that p53 Ser15-P can be used as prognostic factor for HCC, unlike p53 expression. In addition, our data also showed that PCNA correlated with 5-year survival.

Actually, PCNA was already used as one of HCC prognostic factors [24, 25]. Thus, we evaluated relation between p53 Ser15-P and PCNA. In our data, interestingly, p53 Ser15-P did not correlate with 5-year survival in PCNA-negative population while p53 Ser15-P significantly correlated with 5-year survival in PCNA-positive population. Therefore, our data suggest p53 Ser15-P as prognostic factor in the PCNA-positive HCC. There are many pathways that regulate phosphorylation of p53 at serine 15. However, it is still question why p53 Ser15-P affected HCC patient's survival only in PCNA-positive patients even though we clearly showed that p53 Ser15-P significantly correlated with 5-year survival in PCNA-positive population.

## 5. Conclusions

In conclusion, our analysis demonstrated that patients who were PCNA-positive and p53 Ser15-P–negative showed significantly worse survival outcomes than patients who were PCNA-positive and p53 Ser15-P–positive. Therefore we can monitor p53 Ser15-P for identifying poor survival group in PCNA-positive patients, and this might be helpful for prognosis and treatment of HCC especially in PCNA-positive patients.

## Figures and Tables

**Table 1:** Demographics and tumor characteristics of 199 HCC patients.

Variables patients, *n*	Total cohort (n = 199)
Mean age (years)^a^	55 ± 7.99
Gender (Males/Females)	160/39
Patients (HBV/HCV/other)	146/10/43
Median level of serum AFP (ng/ml)	39.6
Cirrhosis (presence/absence)	97/102
Tumor stage (T1/T2/T3)^b^	82/84/33
Vascular invasion (yes/no)	116/83
Major portal vein invasion (yes/no)	19/180
Intrahepatic invasion (yes/no)	33/166
Recurrence (yes/no)	120/79

AJCC TNM staging for HCC; T1, solitary tumor without vascular invasion; T2, solitary tumor with vascular invasion or multiple tumors; T3, multiple tumors or tumors involving a major branch of the portal or hepatic vein.

^a^Mean ± standard deviation; ^b^T1, T2, and T3 grades were judged using the AJCC^6th^ standard.

**Table 2:** Frequency of PCNA, p21, p53, p53 Ser15-P, and p53 Ser392-P expression and scoring of p53 Ser15-P.

Patient status (n = 199)	n	%
PCNA status (cut-off 10%)		
Positive (>10%)	151	75.9
Negative (<10%)	48	24.1
p21 status (cut-off 1%)		
Positive (>1%)	71	35.7
Negative (<1%)	128	64.3
p53 status (cut-off 1%)		
Positive (>1%)	135	67.8
Negative (<1%)	64	32.2
p53 Ser392-P status (cut-off 1%)		
Positive (>1%)	30	15.1
Negative (<1%)	169	84.9
p53 Ser15-P status (cut-off 1%)		
Positive (>1%)	94	47.2
Negative (<1%)	105	52.8
p53 Ser15-P status by percentage of positive cells^a^		
Score 0 (<1%)	105	52.8
Score 1 (1-4%)	48	24.1
Score 2 (5–19%)	40	20.1
Score 3 (20-69%)	5	2.5
Score 4 (70-100%)	1	0.5

^a^Scoring of HCC patients based on standard in Figure 1; n, number of cases; %, percentage of cases.

**Table 3:** Correlation between p21, p53, and p53 serine-P expression in HCC.

		p21	p53	p53 Ser15-P	p53 Ser392-P
	Correlation	1	0.191^*∗∗*^	0.309^*∗∗*^	0.018
p21	coefficient				
	*P* value^a^		0.007	≤0.001	0.801

	Correlation	0.191^*∗∗*^	1	0.157^*∗*^	0.485^*∗∗*^
p53	coefficient				
	*P* value^a^	0.007		0.027	≤0.001

	Correlation	0.408^*∗∗*^	0.257^*∗∗*^	0.239^*∗∗*^	0.100
PCNA	coefficient				
	*P* value^a^	≤0.001	≤0.001	0.001	0.162

Spearman's rank correlation coefficient and correlation significance of the proteins. Protein expression score was used to calculate Spearman's rank correlation coefficient and significance; correlation coefficient, Spearman rank correlation coefficient; *P* value, Spearman correlation; *∗*, *P* < 0.05 (significant correlation); *∗∗*, *P* < 0.01 (highly significant correlation). ^a^Spearman's rank correlation test.

**Table 4:** Correlation between p53, p53 serine-P, PCNA, and clinicopathological factors.

		Vascular invasion	Major portal vein invasion	Intrahepatic invasion	5-Year survival
	Correlation	0.130	0.142^*∗*^	0.136	0.063
p53	coefficient				
	*P* value^a^	0.067	0.046	0.056	0.373

	Correlation	0.082	0.116	0.152^*∗*^	0.011
p53 Ser 392-P	coefficient				
	*P* value^a^	0.251	0.103	0.032	0.873

	Correlation	-0.010	-0.037	-0.070	-0.161^*∗*^
p53 Ser 15-P	coefficient				
	*P* value^a^	0.888	0.599	0.323	0.023

	Correlation	0.211^*∗∗*^	0.221^*∗∗*^	0.259^*∗∗*^	0.202^*∗∗*^
PCNA	coefficient				
	*P* value^a^	0.003	0.002	≤0.001	0.004

	Correlation	0.331^*∗∗*^	0.356^*∗∗*^	0.405^*∗∗*^	1
5-Year Survival	coefficient				
	*P* value^a^	≤0.001	≤0.001	≤0.001	

Spearman's rank correlation coefficient and *P *value between protein expression score and clinicopathological factors. Correlation coefficient, Spearman's rank correlation coefficient; *P *value, Spearman's correlation; *∗*, *P* < 0.05; *∗∗*, *P* < 0.01. ^a^Spearman's rank correlation test.

**Table 5:** Cox proportional hazard model analysis of p53, p53 Ser 392-P, p53 Ser 15-P, PCNA, age, sex, and clinicopathological factors.

	Exp(B)^a^	Significance^a^	Cl (95.0%)^a^
p53	0.904	0.398	0.716-1.142

p53 Ser 392-P	0.925	0.696	0.627-1.365

p53 Ser 15-P	0.614	0.013*∗*	0.418-0.902

PCNA	1.608	0.002*∗∗*	1.192-2.169

Age	1.093	≤0.001*∗∗*	1.045-1.143

Sex	1.085	0.853	0.456-2.584

Vascular invasion	3.977	0.006*∗∗*	1.484-10.658

Major portal vein invasion	6.289	≤0.001*∗∗*	2.744-14.412

Intrahepatic invasion	3.823	≤0.001*∗∗*	1.970-7.420

Hazard ratio and significance in Cox proportional hazard model analysis. Hazard ratio, Exp(B); Exp(B) < 1 (hazard goes down when factor goes up), Exp(B) >1 (hazard goes up when factor goes up); 95% CIs lower & upper values; significance; *P value; ∗*, *P* < 0.05 (significant correlation); *∗∗*, *P* < 0.01 (highly significant correlation). ^a^Cox proportional hazard model test.

## Data Availability

The table and figure data used to support the findings of this study are included within the article.

## Abbreviations

AFP: *α*-fetoprotein
HATs: Histone/lysine acetyltransferases
HBV: Hepatitis B virus
HCC: Hepatocellular carcinoma
HCV: Hepatitis C virus
IHC: Immunohistochemistry
OS: Overall survival
p53 Ser15-P: p53 TAD phosphorylation at Ser15
p53 Ser392-P: p53 TAD phosphorylation at Ser392
PCNA: Proliferating cell nuclear antigen
ROS: Reactive oxygen species
RT: Room temperature
TAD: Transactivation domain.
